# Understanding the Role of Maternal Diet on Kidney Development; an Opportunity to Improve Cardiovascular and Renal Health for Future Generations

**DOI:** 10.3390/nu7031881

**Published:** 2015-03-12

**Authors:** Ryan James Wood-Bradley, Sanna Barrand, Anais Giot, James Andrew Armitage

**Affiliations:** 1 School of Medicine, Deakin University, Waurn Ponds, Victoria 3216, Australia; E-Mails: r.woodbradley@deakin.edu.au (R.J.W.-B.); s.barrand@deakin.edu.au (S.B.); 2 Insitut Politechnique LaSalle Beauvais, 60026 Beauvais Cedex, Picardie, France; E-Mail: g.anais@deakin.edu.au; 3 Department of Anatomy and Developmental Biology, Monash University, Clayton, Victoria 3800, Australia

**Keywords:** developmental programming, kidney development, maternal diet

## Abstract

The leading causes of mortality and morbidity worldwide are cardiovascular disease (high blood pressure, high cholesterol and renal disease), cancer and diabetes. It is increasingly obvious that the development of these diseases encompasses complex interactions between adult lifestyle and genetic predisposition. Maternal malnutrition can influence the fetal and early life environment and pose a risk factor for the future development of adult diseases, most likely due to impaired organogenesis in the developing offspring. This then predisposes these offspring to cardiovascular disease and renal dysfunction in adulthood. Studies in experimental animals have further illustrated the significant impact maternal diet has on offspring health. Many studies report changes in kidney structure (a reduction in the number of nephrons in the kidney) in offspring of protein-deprived dams. Although the early studies suggested that increased blood pressure was also present in offspring of protein-restricted dams, this is not a universal finding and requires clarification. Importantly, to date, the literature offers little to no understanding of *when* in development these changes in kidney development occur, nor are the cellular and molecular mechanisms that drive these changes well characterised. Moreover, the mechanisms linking maternal nutrition and a suboptimal renal phenotype in offspring are yet to be discerned—one potential mechanism involves epigenetics. This review will focus on recent information on potential mechanisms by which maternal nutrition (focusing on malnutrition due to protein restriction, micronutrient restriction and excessive fat intake) influences kidney development and thereby function in later life.

## 1. Maternal Nutrition and the Kidney

A plethora of evidence exists for to support the crucial role that the environment plays during development in determining the future health of the offspring. *Developmental programming* refers to the mechanism underlying this phenomenon and is defined as “a permanent change in the structure or function of an organism due to alterations in development that occur in response to an environmental stimulus during critical periods of organ development” [1]. Particularly implicated is maternal nutrition, which has been shown to have an effect on offspring health, with both over- and under-nutrition having negative consequences on offspring health [2,3,4,5]. A number of studies have provided evidence to support the hypothesis that maternal nutritional status plays a critical role on the health and wellbeing of the fetus [6,7,8]. Changes to maternal nutritional status may change the ability of the mother to supply the growing fetus with the required nutrients for optimal growth. Initial studies during early and mid 20th Century documented the nutritional requirements during pregnancy in humans [9,10] as well as animals [11,12,13,14], indicating the importance of a diet containing sufficient amounts of essential nutrients (proteins, fats, carbohydrates) and micronutrients (iron and calcium) and a sufficient caloric value. These studies reported that protein or micronutrient restriction in the mother lead to poor offspring growth *in utero* and in early postnatal life. It was much later that we began to appreciate the biological consequences of these fetal trade-offs in response to a sub-optimal maternal diet.

Perturbations to the maternal diet during pregnancy and/or lactation are associated with increased risk of renal, cardiovascular and metabolic disease in the offspring [15,16]. The developing kidney is particularly susceptible to the effects of a suboptimal maternal diet; changes in expression of genes and proteins important for kidney development, are likely to underlie the developmental programming of kidney structure and physiology. This paper will review the literature to describe the role of maternal macro- and micro-nutrient imbalance in influencing fetal and neonatal kidney development, final nephron complement and the effect of these structural abnormalities on adult function. The mechanisms underlying these observations will also be discussed.

In order to understand developmental programming of renal structure and function in humans, animal models using dietary interventions have been established. This is primarily due to the difficulty in quantifying structure of the human kidney by non-invasive methods. For example, the effects of maternal dietary zinc, vitamin A and protein restriction or maternal dietary fat excess all have been shown to have a negative impact on kidney development and later renal disease in the offspring [16,17,18,19]. Furthermore, a decrease in nephron number is hypothesized to lead to disrupted fluid and electrolyte balance, which in turn, may lead to volume expansion and finally glomerular hyperfiltration and systemic hypertension [20]. Importantly, restricted maternal protein intake in pregnancy has been linked to reduced birth weight, impaired nephron endowment, elevated blood pressure and reduced glomerular filtration rate (GFR) [21,22,23,24,25,26,27,28,29].

### 1.1. Maternal Protein Restriction Programs Reduced Nephron Number but the Physiological Consequences are Unclear

Interestingly, despite there being a consistent observation from several research groups that protein restriction during development results in a reduction in nephron number, the follow-on effects on renal function (characterized by a measurement of glomerular filtration rate; GFR) and blood pressure are far from clear due to inconsistent techniques used. For example, early studies by Woods *et al.* [26] clearly showed that male offspring from protein restricted dams had a 25% nephron deficit, an 11% GFR reduction and a 10 mmHg increase in mean arterial blood pressure compared with control rats. The calculated single nephron glomerular filtration rate in these protein deprived offspring was 20% greater in protein-restricted than in protein-replete controls [26]. Increases in individual nephron GFR can be an indication of renal hyperfiltration, which can ultimately lead to further nephron loss, perpetuating this cycle [20].

Later studies demonstrated that the phenotype induced by maternal protein-deprivation is not invariant. Woods *et al.* [27] reported that maternal diet affected offspring in a sex-specific manner; female offspring of protein restricted *Sprague-Dawley* rats did not demonstrate hypertension or a nephron deficit. This suggests that sex-dependent variations in developmental programming of the kidney exist, are subtle and highly reliant on the concentration of dietary protein or, potentially, timing of the intervention. Furthermore, reductions in nephron endowment associated with intrauterine protein restriction, are not always accompanied by hypertension. For example, Zimanyi *et al.* (2006) reported a nephron deficit in *Wistar-Kyoto* (WKY) rats fed an 8.7% (low) protein diet (control rats were fed a 20% protein diet) with no alteration in blood pressure or evidence of renal hyperfiltration in the protein-restricted group [30]. Maternal low-protein or caloric restricted diets induce low birth weight in the offspring [31,32] and this reduction in body size may correlate with changes in cardiovascular function and growth [33,34]. Catch-up growth may exacerbate the adverse consequences, such as hypertension, of developmental programming [15,35,36]. Together, these data suggest that maternal protein restriction during pregnancy most likely results in altered kidney and morphology but the follow-on effects on physiology and function are currently not well understood. The variability in phenotype may be resultant from exposure to dietary insult at different points of kidney development. Knowledge of the molecular processes involved in controlling kidney development and ultimately adult morphology are essential for understanding the impact of maternal protein restriction and will be discussed in further detail later in the review.

### 1.2. Maternal Overnutrition Has Subtle Impacts upon Kidney Development and Function in the Offspring

Although early programming studies focused on the role of maternal undernutrition as models for the Dutch Hunger Winter studies [37,38,39], it is apparent that overnutrition is now a major risk to human health and wellbeing. The concept of maternal malnutrition must therefore be expanded to include excess caloric or fatty acid intake [40,41,42,43,44]. Over nutrition and obesity during pregnancy is risk factor for poor outcomes for offspring health, including the susceptibility to chronic diseases [45,46] as well as mortality from these diseases [42,47]. Investigating neonatal outcomes in obese mothers, Catalano *et al.* [48] report that maternal obesity leads to fetuses with greater fat mass, and insulin resistance. Likewise, Boney *et al.* [49] reported that children born large-for-gestational-age to obese mothers were at a greater risk of developing metabolic syndrome. In a rabbit model of maternal high-fat feeding (containing 13% fat), Prior *et al.* [50] report greater mean arterial pressure (6 mmHg), heart rate (13 bpm) and renal sympathetic nerve activity (1.3 nU) compared with controls. These animals were slightly heavier throughout postnatal life and at 4 months of age had significantly heavier white adipose tissue (64%) compared with controls. Similarly, Jackson and colleagues report glomerular sclerosis and reduced kidney function (increased urine albumin excretion) in offspring exposed to a maternal diet high in fructose and fat compared to controls [51]. In a rat model of maternal high fat feeding, Armitage *et al.* (2005) report no change in nephron endowment in offspring of fat fed dams compared with controls, but these same high fat offspring had abnormal renal renin activity which the authors hypothesized to underlie the increased mean arterial pressure [52].

The mechanism by which maternal over nutrition leads to poor health outcomes is not fully understood. Obesity is linked to a chronic inflammatory state in both the mother and fetus [53] and it is possible that this is mediating the changes in gene expression and the development of the offspring.

### 1.3. Maternal Micronutrient Status and Its Impact on Offspring Kidney Health

*In utero* development is characterized by rapid cell synthesis and therefore the developing fetus has a particularly high demand for nutritional substrate. Micronutrients are critical co-factors in enzymatic pathways of cell synthesis in the fetus and therefore there is a high dietary demand for these compounds during pregnancy. Maternal deficiencies in micronutrients can have lasting detrimental effects on the fetus and again there is evidence that the kidney is preferentially affected. Lelievre-Pegorier *et al.* [54] report a 20% reduction in nephron number in adult rats born to mothers that were fed a vitamin A deficient diet during 3 weeks prior to pregnancy, throughout pregnancy up until day 21 of gestation. The mechanism underlying this abnormal kidney phenotype is most likely the fact the vitamin A is a key ligand for the cRET receptor, which controls the earliest processes of kidney development (see Section 2 for full details).

Although the role of vitamin A is well established, other maternal micronutrient deficiencies are associated with abnormal kidney structure or function in her offspring; but with less clear mechanisms Lisle *et al.* [55] reported that diet-induced maternal iron deficiency is associated with greater systolic blood pressure and reduced glomerular number in adult offspring, when compared with controls. Similarly in a model of maternal zinc restriction, Tomat and colleagues [17,56,57] reported reduced nephron number and kidney function (determined by nitric oxide synthase activity) as well as greater blood pressure (tail cuff plethysmography) when compared with controls. The pathway by which different micronutrient deficiency leads to similar morphological phenotypes remains unknown; zinc deficiency has negative effects on branching morphogenesis; a process key to kidney development, but can also impact upon the development of the placenta, thereby affecting the growth potential of the entire embryo or fetus [58].

Given the great disparity in the forms of maternal dietary challenge it is intriguing that a similar phenotype is observed in the offspring in response to challenges as diverse as zinc or protein deficiency. This prompts the hypothesis that fundamental processes in kidney development may be sensitive to an array of abnormal signals from the environment, eliciting a common adaptive response and resulting in a characteristic phenotype. In order to discuss these mechanisms, we will first consider the factors that underpin the development of the kidney.

## 2. Kidney Development

### 2.1. Early Signaling: Specification of the Ureteric Bud and Metanephric Mesenchyme

**Figure 1:**
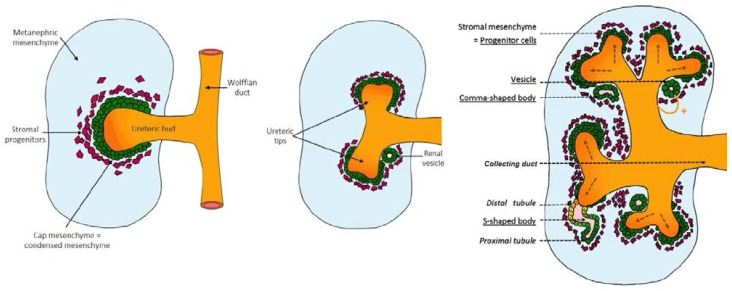
Kidney development. The mammalian kidney develops through three stages (pronephros, mesonephros and metanephros). The pronephros develops from the nephric duct, following which, mesonephric development occurs in conjunction with the degeneration of the pronephros and the formation of the mesonephric tubules. The metanephros develops from the induction of the metanepheric mesenchyme by the ureteric bud from the nephric (Wolffian) duct. As the ureteric bud branches into the metanephric mesenchyme, the mesenchyme around the tips of this branching structure are induced to form renal vesicles. Renal vesicles will progress through the comma-shaped body and S-shaped body stages before connecting to the collecting system of the developing kidney to form a developed nephron.

The kidneys play a vital role in health and disease. In mammals, the kidney develops through three successive stages (Figure 1):

The Pronephros, a rudimentary structure that develops from the lateral plate and intermediate mesoderm and continues to develop into the mesonephros [59].

The Mesonephros, a transient organ, although the mesonephric (nephric) duct is important for the development of the metanephros, the permanent mammalian kidney [59].

The Metanephros which begins to develop at day 35 in human gestation, embryonic day 11.5 (E11.5) in the mouse [60] and embryonic day E12–E13 in rats [61,62].

Nephrogenesis is complete by 36 weeks gestation in humans, by about PN3 (postnatal) in mice [59,63] and PN7 in rats [64]. The metanephros begins to develop when cells from the caudal end grow out from the Wolffian duct (or mesonephric duct) forming the ureteric bud in response to growth factors (discussed in the following sections) secreted by the metanephric mesenchyme [59,64,65,66]. Under the influence of these growth factors, the ureteric bud invades the metanephric mesenchyme and branches forming the collecting duct system.

### 2.2. Genetic Control of Kidney Development

#### 2.2.1. Control of Branching: Molecules Involved in Branching and Inhibiting Lateral Branching

**Figure 2:**
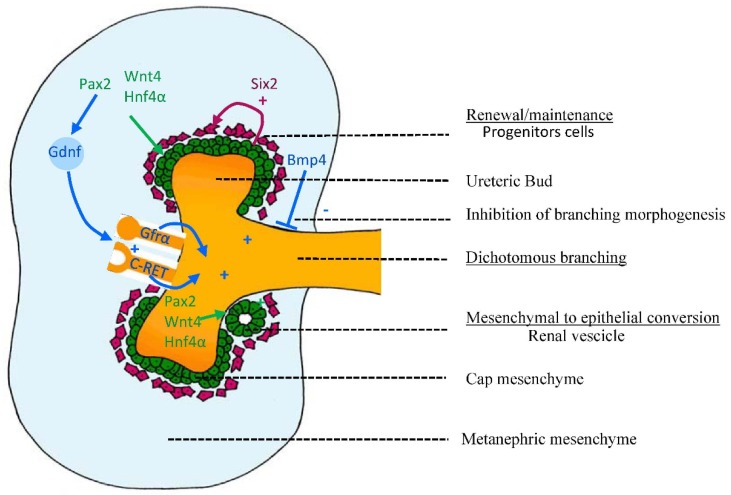
Molecular control of early kidney development. Early development of the metanephros, the permanent mammalian kidney, is under tight molecular control. Outgrowth and branching morphogenesis of the ureteric bud is controlled by the expression of receptors *Gfrα1* and *C-ret* to which *Gdnf* binds. Production of *Gdnf* is under the influence of *Pax2*, Together these factors stimulate branching morphogenesis of the kidney. *Bmp4* inhibits lateral branching of the ureteric, which ensures tight control of branching morphogenesis. Induction of the metanephric mesenchyme to undergo epithelial to mesenchymal transition is influenced by *Wnt4* and *Hnf4α*. The formation of renal vesicles (from the cap mesenchyme) is controlled by the number of *Six2* positive cells (denoting renal stem cells). Once committed to forming a nephron, these cells begin to express *Pax2*, *Wnt4* and *Hnf4α*.

Branching morphogenesis is the dichotomous arborisation of ureteric epithelium and is responsible for the development of the collecting duct system of the kidney. The process of branching morphogenesis is tightly regulated by inhibitors (BMP4, bone morphogenic protein 4), promoters (GDNF, glial derived neurotrophic factor and c-RET, c-ret tyrosine kinase receptor) and growth factors (TGF, transforming growth factor β superfamily). These genes are essential regulators of ureteric bud branching and patterning [67] (Figure 2).

Branching morphogenesis begins with the interaction between the metanephric mesenchyme and the ureteric bud, whereby inductive signals from both embryonic derivatives (GDNF is released from the metanephric mesenchyme and binds to its receptors c-RET and GFRα1 at the tips of the ureteric bud) initiate the branching process [68,69,70,71,72]. Tight regulation of branching morphogenesis is essential to both kidney development and function.

#### 2.2.2. Control of Nephrogenesis: Epithelial-Mesenchymal Transition (EMT)

Nephrogenesis is intricately involved with branching morphogenesis. The induction of nephron development occurs only at the tips of the developing ureteric tree. Metanephric mesenchymal cells adjacent to the ureteric branch tips are induced, leading to condensation, epithelialization and proliferation of the cap mesenchyme. This induced mesenchyme expresses genes involved in extracellular matrix formation and remodeling, cell-adhesion molecules, cell survival and proliferation [73,74,75]. Under influence of the genes *Pax2*, *Wnt4* and *Hnf4α* [76,77,78,79] the induced mesenchyme undergoes epithelialisation and differentiates successively into renal vesicles, comma-shaped bodies, S-shaped bodies before the glomerular vascular tuft develops and attaches to the proximal tubule to finally form mature nephrons, the functional units of the kidney.

## 3. Specification of Nephron Structures

The induced mesenchyme continues to develop through various morphological steps including a comma-shaped body which is characterised by expression of the primitive podocyte marker MNFB and genes involved with *Bmp* signalling including *Bmp2*, *4*, *7* [80,81,82,83,84]. Then, the comma-shaped body develops into an S-shaped body through FGF8 and PAX2 signalling [77,85,86], linking up with the ureteric epithelium. The S-shaped body contains the primitive cells of the glomerulus with the invasion of angiogenic cells. With capillarisation and connecting to the tubule (influenced by FGF2 and LIF expression) [87] the basic structure of the nephron is complete. However, the nephron continues to undergo maturation in the form of tubular elongation.

The metanephric mesenchyme is preserved through the maintenance of a population of progenitor cells. This leads to sufficient support of the progression and development of nephrogenesis. Extensive study, aimed at identifying the genes involved in the maintenance of this cell population, has indicated the importance of Six2, Fgf2 and Bmp7 [88,89]. Self *et al*. [90] reported that knockout of Six2 lead to premature development of renal vesicles. This suggests that Six2 is required to maintain a population of cells required for the development of progenitor cells that form renal vesicles. The generation of Six2 positive cells is related to cortical stromal cells expressing Foxd1, which suggests that there are complex interrelationships between cell subsets that give rise to cells that can control the development of structures within the kidney [91].

## 4. Arcade Formation

In humans nephrogenesis is complete by approximately 36 weeks of gestation, while in rodents, nephrogenesis continues for a short time after birth [64,92]. Until approximately 14–15 weeks of pregnancy in humans, and the end of gestation in rodents, each tip of the ureteric tip connects to a single nephron produced by the surrounding mesenchyme. When arborisation of the ureteric tree is finished and branching ceases, nephrogenesis continues by a process of nephron arcade formation whereby multiple nephrons attached to the same tip.

A second nephron is induced by the same tip, attaches to this tip and the connecting tubule of the older nephron shifts to the new nephron. Other nephrons are then induced until four to seven nephrons form an arcade arrangement by being attached to the stalk rather than just to the tips forming a common connecting segment. Arcade formation enables deep and mid-cortical nephrons to connect to collecting ducts located in the renal cortex [93]. After 20–22 weeks of gestation in humans, and by the very end of kidney development in rodent, nephrons are attached individually and directly along the entire length of the collecting duct and are not incorporated in the arcade [63,65]. Arcade formation is thought to be underpinned by the activity of a number of genes within the Notch pathway as they are involved in the formation of intercellular connections (such as N-cadherin and integrins), however further elucidation of the mechanism is required [94]. Arcade formation is a period of rapid nephron formation and therefore disruption of kidney development durign this critical window can have a profound impact.

## 5. Final Nephron Endowment

The processes controlling the time at which nephrogenesis ends are less well described than those controlling active nephrogenesis. One hypothesis is that the population of cells in the metanephric mesenchyme limits the magnitude of cells that can aggregate and differentiate into nephrons. Indeed this has been proposed as a factor underlying the large variability in nephron number in mammals [95,96,97]. In human, the peak period of nephrogenesis occurs during the last third of pregnancy after the cessation of branching morphogenesis. The progenitor cells of the metanephric mesenchyme provide the basis from which all the cells of the nephron will differentiate. For this reason, as the population of metanephric mesenchyme diminishes there is a natural cessation of nephrogenesis.

Nephron endowment is also dependent upon the branching capacity of the ureteric bud, as nephrons only form adjacent to ureteric tips [54,64,98]. Retarded branching of the ureteric epithelium can therefore manifest in a permanent reduction of nephrons and impaired kidney growth.

In addition, factors involved in the ureteric branching and the epithelisation of the metanephric mesenchyme including growth factors and their receptors, degrading enzyme, proto-oncogenes, transcription factors, suppressor genes extracellular matrix, teratogens and fetal/maternal environment are all involved in the regulation of kidney development and can impinge on nephron endowment [61,99]. It is perhaps not surprising therefore that the number of nephrons in human kidneys varies widely, from 300,000 to nearly 2 million [95,100,101,102,103,104,105,106].

### Early Investigations Supporting Changes during Kidney Development (Genes and Pathways, Cells and Timing)

Kidney development occurs across a number of periods of plasticity and is therefore subject to a number of “critical periods” when external insults may produce life-long structural and potentially functional changes in kidney. Importantly, once kidney development is complete (at around gestational week 38 in humans and postnatal day 8 in rat), no further nephrons are formed. Therefore, a reduction in nephron number caused by interventions during kidney development persists throughout life and may contribute to the burden of disease in later life. Singh *et al.*, 2007 showed in rats that glucocorticoid exposure during the early stages of kidney development (embryonic day E14 to E15), permanently decreased nephron number at PN30 [98]. This is in agreement with an earlier study by Woods *et al.* (2004), which showed that maternal protein restriction during the second half of the pregnancy, when nephrogenesis is active, is associated with a reduction in nephron endowment [28].

Interestingly, the dietary insult need not occur during the period of active nephrogenesis to promote a reduction in nephron number. Welham *et al.* 2005 demonstrate that exposure low protein maternal diet from start of the pregnancy until day E13 in rats (prior to the onset of nephrogenesis) is sufficient to cause reduction in glomerular numbers by approximately 20% [99]. Additionally, uteroplacental insufficiency and reduction in litter number has similar effect during the late stage kidney development [107,108]. Conversely, removing dietary insult during postnatal life may improve glomerular numbers [107,109] owing to the fact that nephrogenesis in rats proceeds until approximately PN7-8. These studies together suggest that dietary insults during critical developmental window may cause changes, especially, in kidney morphology, which may translate to physiological deficits.

The underlying nephron deficit observed in studies of maternal dietary manipulation must be underpinned by changes in the expression of genes and proteins. However, only few studies have analyzed the early gene expression profiles of the kidney exposed to a suboptimal intrauterine environment and how these changes lead to nephron deficit still remains unclear. Abdel-Hakkem and colleagues (2008) induced global caloric restriction in *Sprague-Dawley* rats, and analyzed 10 critical genes involved in branching morphogenesis and nephrogenesis at E20 [110]. Genes involved in mesenchymal to epithelial transformation (*Wnt4*, *Wt1*, *Fgf2* and *Bmp7*) were found to be up-regulated, while genes involved in branching morphogenesis (*Pax2*, *Gdnf*, *Fgf7*, *Bmp4*, and *Wnt11*) were down-regulated [110]. Additionally, proteins of GDNF and MAPK-ERK pathway (e.g., Gfraα1, phosphorylated ERK1/2 and mitogen-activated protein kinase 1/2) are altered already at E20 by maternal food restriction, indicating the importance of these pathways for normal nephrogenesis [111]. In addition a recent study in rats, adult offspring (16 weeks) of low-protein dams showed up-regulation of TGF-β1 and ZEB2 mRNA in kidneys displaying nephron deficit [112]. The authors further suggested that TGF-β1 may induce ZEB2 expression further leading to down-regulation of specific miRNAs within the glomeruli, leading to altered function [112]. These studies suggest that aberrant gene expression of various pathways may underlie reduced nephron endowment, however, the exact mechanism remain to be determined.

For some time, there have been suggestions that apoptosis of mesenchymal cells in developing kidneys play a role in the reduction of nephron endowment [113]. Rats exposed to a maternal LP diet demonstrated a 20% reduction in adult nephron number and up-regulation of apoptotic pathways (e.g., Bax and Bcl-2) [99]. Apoptotic gene expression is also altered by maternal undernourishment [114] particularly increased levels of proteins in late embryonic life (E20; Fas-receptor and Caspase-9) and early postnatal life (PN1; Fas-receptor and caspase-3). These molecular changes were accompanied with increased apoptosis in the nephrogenic zone (the area of active nephrogenesis) of the kidney [115]. These findings demonstrated that maternal programming might increase apoptosis in the developing kidney, which in turn may contribute to nephron deficit.

Other mechanisms proposed to contribute to the nephron deficit, include dysregulation of the Renin-angiotensin system (RAS). Angiotensin is essential in forming blood vessels and tubules during nephrogenesis as well as the development of smooth muscle in the ureter [116,117]. Thus, deficits in nephron number may be preceded by suppression of RAS further contributing to disturbed fluid and electrolyte balance (dysfunction of kidney) and adulthood hypertension. Interestingly, a recent report suggests sex-specific alteration of RAS system is induced by *in utero* exposure to a low-protein diet [19]. Cooke *et al.* 2014 showed that expression of Angiotensin converting enzyme (ACE) was up-regulated in male, but not in female offspring, as early as at E19 relative to control [19]. Additionally, expression of the G-protein coupled receptor of RAS, AT1R, was observed in male offspring at PN21 [19]. Thus, maternal low protein diet may alter nephron numbers in the developing kidney through altered expression of the RAS system, especially in the male offspring. Another study has highlighted the potential function of the Na co-transporters and the RAS in the developmental programming of the kidney [109]. Na-K-Cl co-transporter and Na-Cl co-transporters display functional role in Na reabsorption in kidney and altering levels of these co-transporter could ultimately lead to development of adulthood hypertension.

The exact mechanism underpinning the programming of a nephron deficit remains unknown, but most likely involve alterations in multiple pathways such as apoptotic-pathways, RAS and specific EMT related pathways. The mechanism underlying these gene modulations is proposed to be heritable but not related to mutations in the genome.

## 6. Maternal Diet and the Offspring Epigenome

Changed gene expression; not mutations within the genome, but changes in the way that the genome is expressed, is controlled by epigenetic “marks” such as gene methylation, histone modifications and the binding of DNA proteins. These marks can act independently or in concert to modify expression of genes. Epigenetics may play a role in developmental programming of adult disease as it provides a mechanism that explains the change in gene expression, and therefore the change in phenotype. These changes can be brought about through multiple epigenetic pathways.

The maternal environment, before, during and after birth can have a significant impact on the epigenome of the offspring [118,119,120,121,122]. Essentially, the epigenetic profile of the organism is malleable during any stage of development—as during this stage the genome is undergoing modifications and specification. Before the genome becomes heritably stable, changes in the epigenetic profile can be incorporated into the phenotypic profile and therefore be inherited over multiple cellular cycles and alter the gene expression of that cell line or organ system. One of the most well studied models of how the maternal environment affects the offspring genome through epigenetic mechanisms, is that of the *agouti* mouse.

The *agouti* viable yellow (*AvY*) mouse strain has been used extensively to demonstrate the effects of a methyl deficient diet and subsequent disease outcome. The agouti gene is under the control of a methylation sensitive promoter and infers a yellow coat colour and an increased preponderance to obesity, diabetes, cancer and a shorter lifespan when expressed [123,124,125]. Pregnant *AvY* mice exposed to methyl deficient diets give birth to pups displaying variable coat colours but the majority of offspring present with the agouti phenotype. This phenotype occurs in response to hypomethylation of the agouti locus and an increase in expression. The phenotype is not reversible by later methyl donor supplementation. Where methyl donors are freely available in the maternal diet, the agouti locus remains methylated and the gene repressed, thus the majority of offspring have a brown coat and normal physiology, metabolism and life expectancy.

Studies that have investigated the consequences of maternal dietary manipulation on offspring phenotype, commonly report changes in gene expression and major function in the offspring [126]. Epigenetic control of gene expression is maintained and regulated by three major mechanisms: DNA methylation, histone acetylation and non-protein coding RNA [126]. Few studies have investigated the role of epigenetics in kidney development however there is strong evidence that relatively high level (*i.e.*, changes to enzymes that have wide-reaching effects, rather than those that control a particular process of kidney development) epigenetic changes may drive later kidney disease. Pham *et al.* [127] used uteroplacental insufficiency as a model of intrauterine growth restriction and report that CpG methylation of p53 was altered leading to changes in expression of genes involved in apoptosis within the kidney. While methylation is one form of epigenetic modification, there is also the production and implication of microRNAs. The importance of Dicer; the enzyme which cleaves non-coding microRNAs into active microRNAs during mammalian kidney development has been shown by Nagalakshmi *et al.* [128]. These authors used a transgenic mouse model (Six2Cre) where *Dicer* was conditionally ablated from the progenitors of the nephron epithelium. The removal of Dicer lead to elevated apoptosis and premature termination of nephrogenesis. Ablation of *Dicer* from ureteric bud epithelium (using HoxB7Cre transgenic mice) resulted in disrupted branching morphogenesis and the development of renal cysts [128]. Likewise in a mouse model with the conditional knockout of Dicer1 from the developing urogenital tract, Pastorelli *et al.* [129] report renal abnormalities including hydronephrosis and cyst formation. Furthermore, offspring had increased renal apoptosis combined with reduced glomerular number. These results illustrate the importance of miRNA function during kidney development.

Offspring of rats whose mothers were fed protein restricted diets in pregnancy and lactation demonstrate global changes to the methylation status of whole organs, and important gene pathways that play a major role in kidney development and adult function [130,131,132]. These studies have further investigated these effects to show that important enzymes involved in setting the methylation profile of the genome are altered (specifically DNMT1, which is involved in maintaining the methylation status of the genome during replication), as well as receptors involved in protein metabolism [133,134]. Given that methylation is highly dependent on dietary intake of methyl donors, it has now been demonstrated that when either the maternal or early postnatal diet are supplemented with methyl donors (such as glycine and folic acid), the deleterious cardiovascular effects of maternal protein restriction may be at least partially ameliorated or rescued. Song *et al.* 2010, suggest that a number of genes underlying ureteric bud formation (including elements of the renin angiotensin system) are controlled by histone acetylation; certainly HDAC inhibitors have a negative impact on kidney development. Interestingly, despite the intense interest in determining epigenetic changes in models of maternal or developmental programming, there is still little direct evidence for an epigenetic modification in the development of kidney disease or morphological abnormality [135]. This is likely due to the complex interplay between histone acetylation, gene methylation and miRNA post-translational modifications.

## 7. The Impact of Nephron Endowment on Adult Cardiovascular and Renal Health

As nephrons are the functional units of the kidney a reduction in number is theoretically detrimental to kidney function. Low nephron number may result in adult kidney dysfunction including hypervolaemia, electrolyte balance and toxaemia [136] and is implicated in hypertension and renal disease [137].

The mechanism underlying this link between nephron number and cardiovascular/renal function was first described and popularized by Brenner *et al.* [20]. Brenner hypothesised that a reduction in renal filtration surface area (due to either lower nephron number or lower filtration surface area per glomerulus) would lead to limited sodium excretion, secondary hypervolaemia which promotes an increase in glomerular capillary pressure. Over time this excessive pressure results in glomerular sclerosis [20], further reductions filtration surface area a further increase in systemic blood pressure.

There is strong evidence to support the hypothesis that there is an associate between nephron number and blood pressure but due to fact that present methods for estimating human nephron endowment can only be conducted post mortem it is not possible to show causation. Keller *et al.* [105] reported an inverse association between nephron endowment and hypertension in humans. However, it was unclear whether the lower numbers of nephrons in subjects with a history of hypertension were responsible for hypertension or a consequence of hypertension. Keller *et al.* [105] analysed kidneys obtained at autopsy from Caucasian subjects (normotensive and hypertensive patients) between the age of 35 and 59 years, and found that 10 normotensive subjects had a median glomerular number of 1,429,200 while 10 hypertensive subjects had a median glomerular number of 702,379. Although it was unclear whether the lower numbers of nephrons in subjects with a history of hypertension were responsible for hypertension or a consequence of hypertension. The hypertensive patients showed very few obsolescent glomeruli which may suggest that the hypertension had not caused the deficit in nephron number. Douglas-Denton *et al.* [100] also reported that hypertensive subjects have significantly fewer nephrons (746,468 ± 133,240) than normotensive subjects (1,402,360 ± 346,357).

Low birth weight (LBW) is often used as an indication of intra-uterine growth restriction and has been correlated with the incidence of kidney disease [138,139]. For example, Lackland [138] provided evidence that kidney failure and end-stage renal disease demonstrate a racial and geographic disparity. This concept is supported by findings of Hoy *et al.* [101] that the kidneys of Australian Aboriginals contain 30% fewer nephrons than those of non-indigenous Australians. This phenomenon may explain in part why Australian Aborigines in remote areas have a much greater incidence of renal disease (as well as hypertension and cardiovascular disease) than non-Aboriginal peoples in remote areas [140]. A study by Lackland [141] focused on the incidence of hypertension, demonstrating that adults born with LBW had a much higher systolic blood pressure and were more likely to suffer from hypertensive-related end-stage renal disease than those of normal birth weight. Investigating the developing kidney in the human fetus, Lori *et al.* [142] report that IUGR was associated with reduced renal volume compared to gestational-age adjusted controls. While renal volume can be indicative of nephron number, no technique is available for *in situ* measurement of human fetal nephron number. The investigation by Lori *et al.* does provide information on how the human intrauterine environment impacts directly upon the kidney and the finding is repeatable. A more recent investigation by Wang *et al.* [143] in human fetal kidneys also reports that fetal growth restriction is associated with smaller kidneys which appears to be caused by increased apoptosis and reduced expression of renin and angiotensinogen.

Although the limited human data appear to be supportive of an association between low birth weight and reduced kidney size this association is not always observed in animal studies [144] where tighter control over experimental conditions and potential bias are possible. In support of this hypothesis Langley-Evans *et al.* [145] reported that a deficit of 13% in nephron number was associated with a 13 mmHg elevation in arterial pressure of protein-restricted offspring (*Wistar* rats exposed to a 9% protein diet, while controls had a normal 18% protein diet). On the other hand, no relationship between nephron number and blood pressure was found in female offspring from protein restricted *Sprague-Dawley* dams (on a 8.5% protein diet, while controls were on a 19% protein diet) by Woods *et al.* [27].

Investigations into the relationship between nephron number and cardiovascular health do not support the Brenner Hypothesis unconditionally (see review by Kett and Denton [146]. Nephron deficiency is not always associated with elevated arterial pressure. For example, Zimanyi *et al.* (2006) reported a nephron deficit in offspring of *WKY* rats fed an 8.7% (low) protein diet (while control rats were fed a 20% protein diet) but blood pressure and renal filtration were normal [30]. The absence of change in blood pressure between the dietary groups may also be a product of offspring exposed to maternal protein restriction failing to demonstrate catch-up growth.

Moreover, reduced nephron endowment does not necessarily underlie systemic high blood pressure, in a model of hypertension unrelated to maternal nutrition. Investigating the *Spontaneously Hypertensive Rat* (SHR), Black and colleagues [147] report no change in nephron number but a 29 mmHg increase in blood pressure (measured via tail cuff plethysmography). This is in contrast to the findings of Skov *et al.* [148] who reported reduced nephron number in the *SHR* compared to normotensive *WKY* rats. While it is understood that the hypertension that develops in the *SHR* is multifactorial, the relationship that nephron endowment may play is unclear. The results from these studies indicate that the relationship between nephron number and blood pressure is not direct and may be influenced by other factors including the renin-angiotensin-aldosterone systems and aberrant sympathetic nerve activity. The other potential confounding factor is the blood pressure measurement technique. Tail-cuff plethysmography is known to induce a stress response in animals that may lead to increased blood pressure [149,150]. Further studies are required using less stressful techniques, such as radiotelemetry [149,150], to further elucidate the underlying mechanism of hypertension within the *SHR*.

Tonkiss and colleagues, using radiotelemetry devices, reported that malnourished rat offspring (6% maternal LP diet) demonstrated a 4 mmHg increase in diastolic blood pressure compared with controls, however offspring of protein-restricted rats elicited a greater cardiovascular arousal response (increased systolic pressure) to acute stress compared with controls [151]. This same study did not report nephron number in malnourished offspring, but did find that malnourished offspring weighed the same as controls at the time of blood pressure measurement. In a rat model of maternal caloric restriction, Brennan and colleagues reported a nephron deficit and no change in adult blood pressure (recorded using radiotelemetry) [152]. Lim *et al.* [153] report that in rats exposed to maternal protein restriction that while there was no change in mean arterial pressure (consciously measured with indwelling catheters) there was greater filtration of the kidney. Therefore, the question still remains about the relative contribution of postnatal growth and nephron number to adult blood pressure.

Although multiple studies have reported low nephron number in offspring of dams exposed to a protein-restricted diet, the renal physiological effects of this reduction in nephron number have received less attention and findings have been contradictory. Alwasel and Ashton [154] reported no change in GFR in male or female rat offspring exposed to maternal LP diet (no nephron number reported), while Nwagwu *et al.* [23] reported reduced GFR in rat offspring exposed to maternal LP diet (no nephron number reported). Hoppe *et al.* [22] using a model of life long protein restriction in rats reported no change in GFR with a 31% reduction in nephron number.

Sidduque *et al.* (2014) show no association between hypertension and low nephron number in the offspring of Sprague Dawley rats fed 6% protein from 12 days of gestation [109]. Neonates of these dams were cross-fostered and studied. Offspring exposed to a high protein diet (20% w/w) *in utero* and cross-fostered to a mother fed a low-protein (6% protein) diet showed increased blood pressure and expression of the co-transporters after birth relative to control animals (20% protein throughout life) [109]. Surprisingly, these animal maintained similar nephron number and blood pressure compared with controls [109]. On the other hand, animals that were maintained on a protein restricted diet throughout the pregnancy and suckling recorded increased blood pressure, increased expression of renin and ANGII, had reduced nephron number, but did not show alteration in the Na co-transporter expression relative to controls [109]. This study indicates that dietary impact at different time points indeed could affect kidney development through different pathways. In summary, suboptimal maternal diet may alter gene and protein expression in the developing kidney leading to permanent reduction of nephron number but whether or not this results in hypertension is clearly dependent on other, yet to be identified factors.

There is also evidence that pre- *vs*. post-natal nutritional planes have differing effects on kidney development in altricial species such as rats and mice. Jennings and colleagues used a cross-fostering method in rats to compare the contributions of the fetal and postnatal environments on offspring growth [155]. Pups that were exposed to protein restriction *in utero* then switched to a control dam post birth underwent catch-up growth and had similar weight to control pups by PN21, while pups that were maintained on a low protein diet postnatally did not. In fact, pups that underwent catch-up growth had reduced longevity. Investigations into catch-up growth also report changes in insulin sensitivity [156] and reduced β-cell proliferation and islet size during fetal life [157]. Wlodek *et al.* [107] using an uteroplacental insufficiency model in rats to induce fetal growth restriction report that offspring that were growth restricted in utero and postnatally had higher blood pressure as well as a nephron deficit, compared to controls. Meanwhile, offspring that were restricted in utero and were then cross-fostered to control dams did not have a reduced nephron endowment or increased blood pressure [107]. These studies indicate that postnatal nutrition of offspring is critical to the development of disease. Many studies that have linked maternal low protein diet to reduced nephron endowment and hypertension also observed accelerated growth postnatally [26,158,159].

The difficulty in translating these rodent studies back to the human condition is that rats and mice are born immature and nephrogenesis proceeds for up to a week postnatal. In humans, this process of nephrogenesis occurs *in utero* until 38 weeks gestation, therefore the postnatal period has little impact upon term infants. In the premature infant, however, the situation may be very different because these children are born whilst nephrogenesis is ongoing. Moreover, in many cases, the supply of breast milk is constrained in mothers of premature infants and formula supplementation may be required.

Investigating the impact of preterm birth in humans on kidney development and function, Gubhaju *et al.* 2014 report that renal function was affected by gestational age and may be linked to renal maturity [160]. Assessing the maturity of preterm kidneys, Sutherland *et al.* (2011) report that while there is a renal maturation in infants born preterm (before the cessation of nephrogenesis) this accelerated maturation is associated with morphologically small glomeruli [161]. These data further illustrate that in both animals and humans there is great potential for the kidney to be influenced by the environment, whether that be maternal or postnatal.

## 8. Conclusions

The dietary intake and nutritional status of pregnant and breastfeeding mothers is a major public health concern given the robust evidence from human and animal studies which indicates that a healthy pregnancy (normal birth weight and absence of congenital defects) predicts a healthy start to life that will continue into the offspring’s adult life. Indeed, maternal malnutrition is still a major health concern world-wide, in both developing nations (malnutrition) and developed nations (over-nutrition). The consequence of poor maternal diets can potentially affect future generations, leading to them developing hypertension, diabetes, obesity or the metabolic syndrome. Of particular interest is the susceptibility of the kidney to the *in utero* environment because this organ develops slowly and across most of fetal life. The molecular control of the kidney has been well-reported (www.gudmap.org), however the impact of maternal diet on these molecular targets not been. It remains to be discovered *when* the maternal diet influences kidney development, *what* molecular processes are effected and *if* the phenotype can be rescued or halted. While the current generation’s *in utero* experience cannot be altered, there are protective measures that can be encouraged to minimise the potential for the *in utero* environment to be deleterious to future health.
